# The Role of NOL3 in Colon Adenocarcinoma Metastasis and Its Association With DNA Methylation

**DOI:** 10.1155/humu/9983517

**Published:** 2025-09-24

**Authors:** Li Hong, Hao Zhang, Ruipeng Wang, Zirui Zhuang, Jinjing Xu, Youyuan Tang

**Affiliations:** Department of General Surgery, The First Affiliated Hospital of Soochow University, Suzhou, Jiangsu Province, China

**Keywords:** colon adenocarcinoma, DNA methylation, immune infiltration, NOL3, prognostic biomarker

## Abstract

Colon adenocarcinoma (COAD) is a particularly aggressive cancer type, frequently identified at a later stage. Thus, it is crucial to enhance our understanding of the fundamental mechanisms that govern COAD cellular behavior and to perform extensive research into the biological foundations underlying its development, progression, invasion, and metastasis. We initially discovered NOL3 as a significant gene influencing COAD metastasis through the application of the weighted gene coexpression network analysis algorithm using the TCGA-COAD dataset. This was succeeded by an assessment of NOL3's expression levels and prognostic significance. Moreover, we investigated the biological roles of NOL3 utilizing transcriptomic data. Our results demonstrate significant associations between NOL3 and immune infiltration in COAD, as well as sensitivity to chemotherapy. Furthermore, we utilized the tumor immune dysfunction and exclusion (TIDE) algorithm to evaluate how various cohorts responded to immune checkpoint therapies. Ultimately, the influence of NOL3 on the metastasis of COAD cells was confirmed through in vitro experiments. Our results indicate that NOL3 can promote COAD metastasis, and its underlying mechanism may be associated with DNA methylation. In summary, NOL3 has been identified by us as a key biomarker for COAD metastasis.

## 1. Introduction

Colon adenocarcinoma (COAD) ranks as one of the most prevalent malignant tumors worldwide, regularly positioned among the Top 3 contributors to deaths associated with cancer [1]. In recent years, there has been a rise in the number of screenings for COAD, and patients frequently receive surgical removal of tumors or a combination of chemotherapeutic agents, leading to a decrease in both incidence and mortality rates [2]. Nevertheless, only 17.7% of individuals with metastatic colorectal cancer live beyond 5 years, and about 25% already present with liver metastasis at diagnosis, while nearly 50% experience liver metastasis within 3 years following their initial surgical intervention. These observations underscore that swift malignant advancement and cancer cell spread are the key factors behind the elevated mortality and diminished survival rates observed in COAD patients [3, 4]. Nowadays, despite recent advancements in the treatment of COAD, including chemotherapy and radiotherapy, the prognosis of COAD remains relatively poor [5, 6]. Therefore, a better understanding of the molecular and signaling mechanisms involved in the progression of COAD metastasis and the development of novel and effective targets is crucial for improving the prognosis of patients with metastatic COAD.

In recent years, notable progress in bioinformatics has considerably enhanced the research and identification of biomarkers related to colorectal cancer. This form of cancer is among the most frequently occurring malignant tumors worldwide, making early detection and tailored treatment essential for boosting patient survival rates. Through the integration of multiplatform transcriptomics data and sophisticated algorithms, scientists have discovered novel biomarkers that are crucial for the early diagnosis and prognosis of colorectal cancer [7]. For instance, one investigation pinpointed specific genes linked to colon cancer by examining RNA-seq data sourced from the Gene Expression Omnibus (GEO) and The Cancer Genome Atlas (TCGA) databases, utilizing machine learning techniques like support vector machines and random forests. These genes demonstrated strong performance in analyzing immunohistochemical image features, successfully differentiating colon cancer patients from healthy controls [8]. In a different study, researchers employed bioinformatics and machine learning strategies to uncover potential metabolic markers associated with colon cancer. These markers are implicated in cancer-related metabolic pathways and exhibit varying degrees of diagnostic accuracy at different stages of the disease [9]. Additionally, the research indicated that a combination of biomarkers, encompassing changes in lipid metabolism and alterations in chemokine levels, can enhance the precision of predicting the prognosis of colon cancer [10]. In conclusion, the integration of bioinformatics offers a novel perspective and methodology for the identification of biomarkers associated with colorectal cancer. These studies not only enhance the diagnostic accuracy of colorectal cancer but also serve as significant references for personalized treatment and prognostic evaluation.

This research focuses on identifying crucial regulatory genes that play a role in the metastasis of COAD and analyzing their functions with the use of the weighted gene coexpression network analysis (WGCNA) approach. We assessed the expression levels and prognostic significance of NOL3 in COAD by leveraging data obtained from TCGA database. Moreover, we investigated the possible functions of NOL3 in COAD through gene enrichment analysis, followed by evaluating the relationship between NOL3 and responses to both immunotherapy and chemotherapy in COAD. Lastly, we investigated the function of NOL3 in COAD cells through in vivo experiments. Collectively, this study systematically elucidates the expression characteristics and regulatory mechanisms of the NOL3 gene in the metastatic process of COAD through the integration of multiomics data analysis, which encompasses gene expression, DNA methylation, and immune infiltration data, alongside in vitro cell functional experiments.

## 2. Materials and Methods

### 2.1. Datasets

Gene expression profiles, clinical information, and DNA methylation data were retrieved from TCGA [11]. We then extracted the data in TPM format and conducted log2(TPM+1) normalization. Ultimately, by keeping samples that contained both RNA-seq data and clinical information, we included a total of 455 tumor samples and 42 normal samples for further analysis.

### 2.2. Gene Enrichment Analysis

In the R programming environment, we employed the Limma package (Version: 3.40.2) to investigate the differential expression levels of NOL3. For TCGA-COAD dataset, the thresholds established for detecting differential expression of NOL3 were “*p* value < 1.3 or fold change < −1.3.” To gain deeper insights into the oncogenic roles of this targeted gene, we examined the KEGG pathways and various components of gene set enrichment analysis (GSEA) related to NOL3 through the ClusterProfiler package in R [12, 13].

### 2.3. Immune Infiltration and DNA Methylation Analysis

To obtain a trustworthy evaluation of the immune scoring outcomes, we employed the CIBERSORT algorithm from the immunedeconv R package to investigate the relationship between NOL3 and immune cell infiltration in COAD [14]. We gathered data on the variations in NOL3 methylation levels between COAD and normal tissues, as well as across different stages, from the UALCAN database [15]. Moreover, insights concerning the expression alterations and associated analyses of methylation probes aimed at NOL3 in COAD were sourced from the SMARP database [16].

### 2.4. Cell Culture

The human normal colon epithelial cells CCD-841 CoN (RRID: CVCL_2871) and NCM460 (RRID: CVCL_0460), along with the human colon cancer cell lines SW480 (RRID: CVCL_0546), SW620 (RRID: CVCL_0547), and HT-29 (RRID: CVCL_A8EZ), were acquired in 2020 from the Cell Bank of the Type Culture Collection of the Chinese Academy of Sciences in Shanghai, China. CCD-841 CoN cells were cultured in DMEM, NCM460 in 1640 medium, SW480 and SW620 cells in L15 medium, and HT-29 cells in McCoy's 5A medium. All media were supplemented with 10% fetal bovine serum. The cells were incubated in a humid environment at 37°C with 5% carbon dioxide. We confirmed that none of the cell lines used were contaminated or misidentified.

### 2.5. Proliferation Ability Analysis

The intervened COAD cells (1 × 10^4^) were cultured in triplicate in 96-well microtiter plates and incubated in an atmosphere of 5% CO_2_ at 37°C. The optical density (OD) values were measured using microplate computer software (BioRad Laboratories Inc., Hercules, CA, United States) according to the protocol provided by the Cell Counting Kit-8 (CCK-8) assay kit (Dojindo, Tokyo, Japan). Subsequently, cell proliferation curves were generated.

### 2.6. Tube Formation Assays

Tube formation assays employed the Matrigel basement membrane matrix (Corning Incorporated, 356234, United States). To begin, an ice-cold pipette tip was utilized to dispense about 40 *μ*L of Matrigel into each well of a 96-well plate, which was then incubated at 37°C to facilitate solidification. Afterward, 1.2 × 10^4^ HUVECs were introduced into the solidified Matrigel. The cells were photographed at 3 h and again at 12 h of incubation using an inverted light microscope (Lecial, Germany).

### 2.7. qRT-PCR

Total RNA was obtained from the cell lines CCD-841 CoN, NCM460, HT-29, SW480, and SW620 using the TRIzol reagent (Thermo Fisher, United States). For cDNA synthesis, 500 ng of RNA was used in conjunction with the HiScript II SuperMix (Vazyme, China). The quantitative RT-PCR was conducted on the ABI 7500 System (Thermo Fisher, United States) with SYBR Green Master Mix. The PCR amplification protocol included 45 cycles, beginning with a 10-min phase at 94°C, followed by 10 s at 94°C and then 45 s at 60°C. GAPDH served as the internal control for this experiment.

### 2.8. Statistical Analysis

The levels of NOL3 expression in normal tissues and COAD tissues were evaluated through the Wilcoxon rank-sum test. For analyzing prognosis, the log-rank test was employed. A *p* value below 0.05 was deemed statistically significant.

## 3. Result

### 3.1. Screening Genes Related to COAD Metastasis

**Figure 1:**
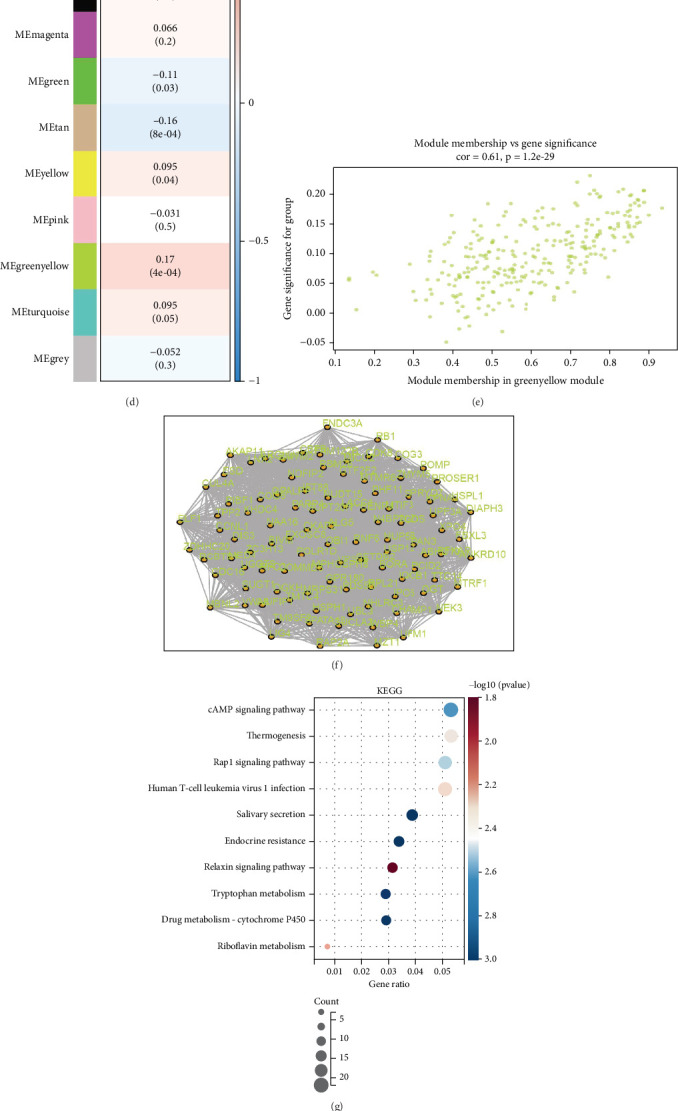
NOL3 is associated with the metastasis of CAOD. (a, b) The ideal soft-threshold power was determined to be 16. (c) The construction of the weighted coexpression network utilized the chosen power values. (d) A heatmap displaying the associations between trait modules is presented. (e) A scatter plot highlights the specific traits linked with the genes in the modules. (f) An interaction network diagram for module genes is shown. (g, h) An analysis of the functions of module genes is provided. (i) A Venn diagram compares module genes with differential.

To pinpoint potential targets that may influence the metastasis of COAD, we divided TCGA-COAD dataset into M0 and M1 stages and then executed a WGCNA. To maintain a scale-free distribution in the network, we identified the optimal power value for the adjacency matrix weight parameter, which was determined to be 16 (Figure 1a,b). Utilizing this power value, we developed a weighted coexpression network model that segmented all genes into 12 unique modules (Figure 1c). By employing the Pearson correlation algorithm, we computed the correlation coefficients and *p* values between module eigengenes and traits, indicating that the greenyellow module showed the strongest correlation, with a coefficient of 0.17 (Figure 1d,e). Additionally, we created an interaction network diagram for the Top 100 genes contained within the greenyellow module (Figure 1f). Using this selection of 100 genes, we performed functional analyses. The outcomes from our KEGG analysis suggested that these genes are linked to pathways, including the cAMP signaling pathway, the Rap1 signaling pathway, and endocrine resistance (Figure 1g). Results from the GO analysis highlighted that these genes are connected to pathways related to cell death, the apoptotic process, and cell localization (Figure 1h). Lastly, we integrated differentially expressed genes from tumor and adjacent normal tissues, as well as prognostic genes sourced from TCGA-COAD dataset, to produce a Venn diagram (Figure 1i). Considering that tumor metastasis is a characteristic of procancer behavior, we focused exclusively on those genes that demonstrated high expression in tumors and acted as prognostic risk factors for COAD patients. Ultimately, NOL3 was identified as the singular key gene associated with metastasis.

### 3.2. Expression and Prognostic Analysis of NOL3 in COAD

**Figure 2:**
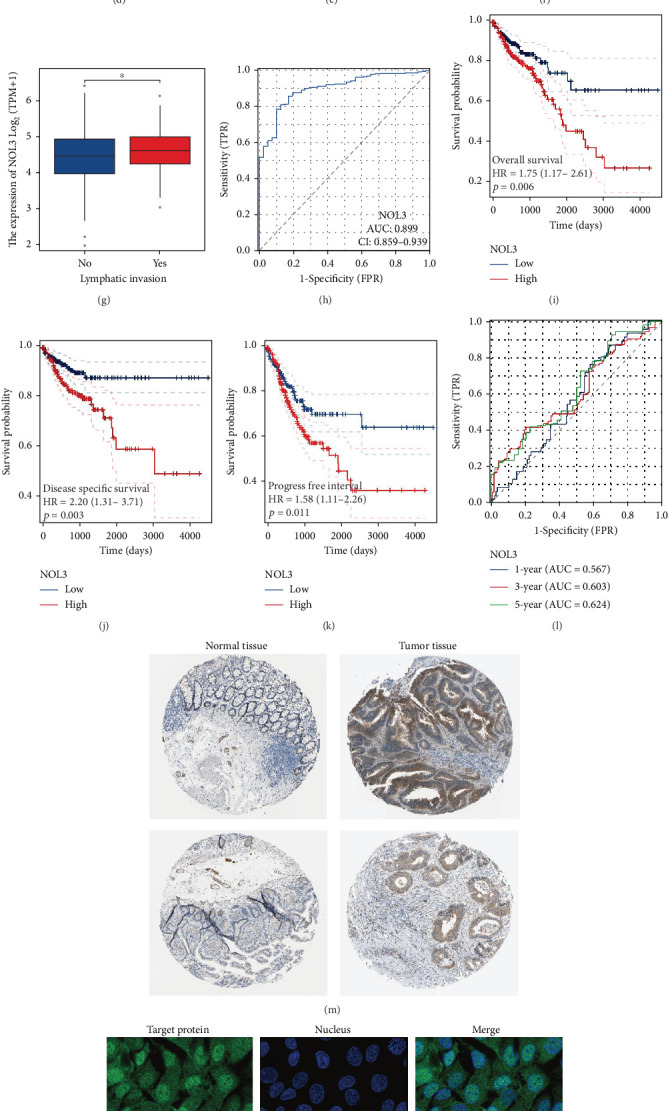
High levels of NOL3 expression are associated with a poor prognosis. (a, b) Differences in NOL3 expression between cancerous tissues and adjacent normal tissues. (c) Variations in NOL3 expression among samples with differing N stages. (d) NOL3 expression differences across samples classified by varying M stages. (e) Comparison of NOL3 expression in samples with distinct T stages. (f) NOL3 expression differences in samples at different clinical stages. (g) Analysis of NOL3 expression in samples with lymph node invasion versus those without. (h) The prognostic significance of NOL3 in COAD diagnosis. (i–l) The prognostic associations of NOL3 in cases of COAD. (m) Differential levels of NOL3 protein in COAD specimens. (n) Analysis of NOL3 protein localization within tumor cells. (o) The differential expression of NOL3 in COAD cell lines. ⁣^∗^*p* < 0.05, ⁣^∗∗^*p* < 0.01, ⁣^∗∗∗^*p* < 0.001.

The analysis of expression variations in NOL3 between paired and unpaired samples from TCGA-COAD dataset revealed that NOL3 is notably upregulated in bladder cancer (Figure 2a,b). Additionally, higher expression levels of NOL3 were observed in samples that exhibited advanced disease with lymph node or distant metastasis; however, expression levels did not show significant differences across various T stages (Figures 2c, 2d, 2e, 2f, and 2g). We also investigated the diagnostic relevance of NOL3 expression in patients with COAD, with the ROC curve suggesting that NOL3 may serve as a significant diagnostic marker (Figure 2h). Moreover, our assessment of NOL3's prognostic relevance in COAD patients indicated that individuals with higher NOL3 expression experienced a worse prognosis (Figures 2i, 2j, and 2k). The findings from the ROC curve further suggest that NOL3 has predictive significance for the prognosis of COAD patients (Figure 2l). Furthermore, analysis via the Human Protein Atlas database reveals that the protein levels of NOL3 in COAD are considerably elevated compared to normal tissues, with NOL3 primarily found localized within the nucleus (Figure 2m,n). Finally, we analyzed the differential expression of NOL3 in COAD cell lines (Figure 2o).

### 3.3. Functional Analysis of NOL3 in COAD

**Figure 3:**
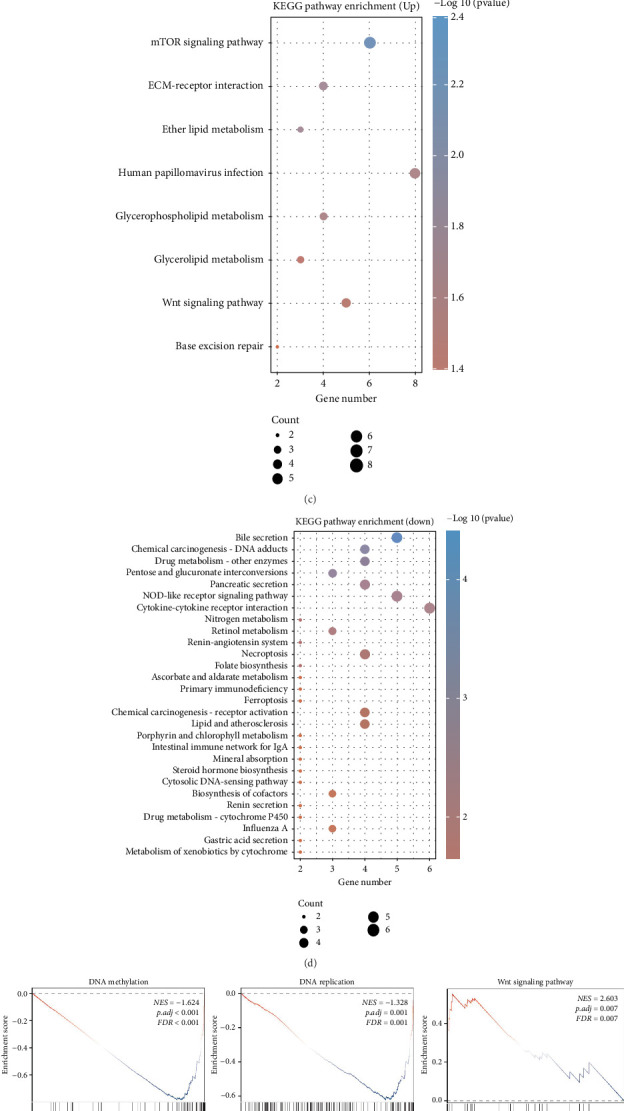
NOL3 plays a significant role in COAD. (a) Maps of volcano plots for the analysis of differences in NOL3. (b) Heatmap illustrating differential expression of genes associated with NOL3. (c, d) KEGG analysis pertaining to genes related to NOL3. (e) GSEA.

In this research, we investigated the role of NOL3 in COAD by exploring its potential involvement through differential analysis. We set specific thresholds to identify differentially expressed genes related to NOL3: *p* < 0.05 and FC > 1.3 or *p* < 0.05 and FC < −1.3 (Figure 3a). To clarify the variations in gene expression between groups with high and low NOL3 expression, we utilized a heatmap (Figure 3b). Additionally, we performed functional enrichment analysis on these differentially expressed genes to confirm the potential significance of NOL3 in COAD. Results from KEGG analysis demonstrated notable correlations between upregulated genes and multiple pathways, such as human papillomavirus infection, mTOR signaling, ECM-receptor interaction, and Wnt signaling (Figure 3c). Moreover, KEGG analysis showed significant connections between downregulated genes and various biological functions, including cytokine–cytokine receptor interaction, necroptosis, lipid and atherosclerosis, and ferroptosis (Figure 3d). Traditional differential analysis utilizing KEGG primarily focuses on comparing gene expression variations between two groups, emphasizing genes that are notably upregulated or downregulated. Nonetheless, this approach may overlook genes that, although not significantly differentially expressed, hold considerable biological importance. Additionally, it does not take into account vital information such as the biological features of genes, interactions within gene regulatory networks, and the functional relevance of the genes themselves. To address these shortcomings, we conducted a more thorough analysis of NOL3 through GSEA. Our results indicate that NOL3 in COAD may be associated with DNA methylation, DNA replication, conditions linked to programmed cell death, and cellular senescence (refer to Figure 3e). Although GO analysis provides detailed classification advantages in biological processes, molecular functions, and cellular components, its categorization remains relatively broad and fragmented. This limitation makes it challenging to directly reflect specific signaling pathways and metabolic networks. Given that this study focuses on the complex phenotype of tumor metastasis, we emphasize a holistic functional interpretation at the pathway level. Therefore, we have selected only the KEGG and GSEA as the core tools for functional analysis in this context. In conclusion, our findings suggest that NOL3 significantly influences COAD, with its potential mechanisms being facilitated through the pathways mentioned above.

### 3.4. Analysis of NOL3 Methylation Levels

**Figure 4:**
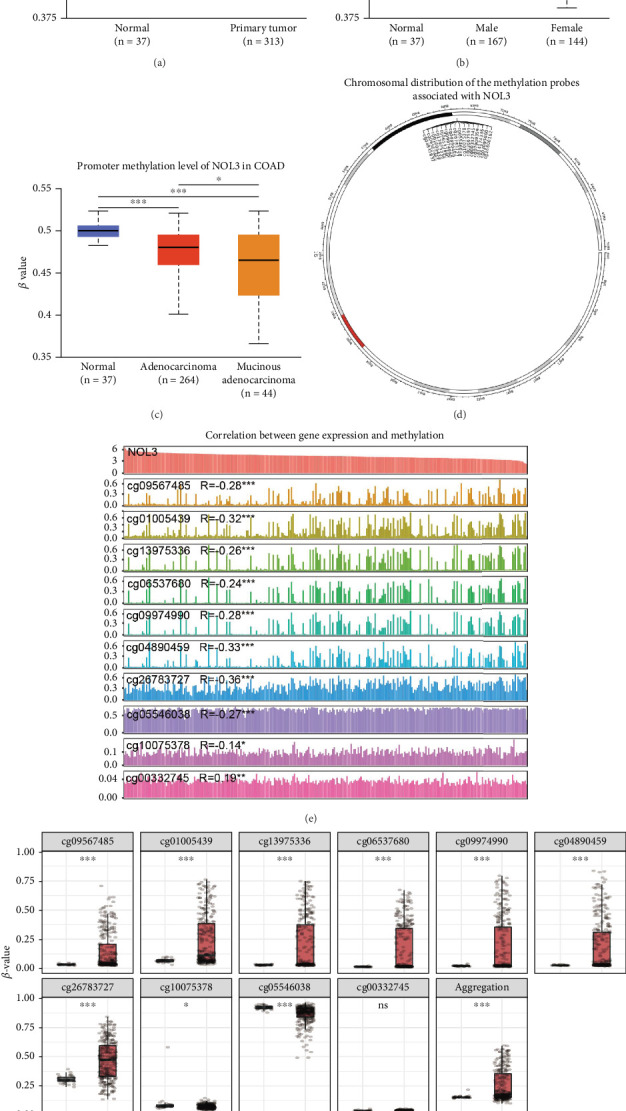
Analysis of methylation correlation for NOL3. (a) A comparison was made of the DNA methylation levels of NOL3 in samples of colorectal adenocarcinoma (COAD) versus normal colon tissues. (b) An analysis was conducted to evaluate the differences in NOL3 DNA methylation levels based on gender. (c) The variations in NOL3 DNA methylation levels across various tumor types of COAD were assessed. (d) The chromosomal distribution of methylation probes associated with NOL3 was analyzed. (e) An investigation was carried out on the relationship between NOL3-associated methylation probes and the expression levels of NOL3. (f) The differential expression of methylation probes related to NOL3 was examined between COAD and normal colon tissue samples. ⁣^∗^*p* < 0.05, ⁣^∗∗^*p* < 0.01, ⁣^∗∗∗^*p* < 0.001.

The analysis of gene enrichment concerning NOL3 unveiled a significant association between NOL3 and DNA methylation in COAD. To investigate this connection further, we executed a detailed study examining the alterations in DNA methylation levels of NOL3 in COAD. Our results indicated that the DNA methylation levels of NOL3 in the COAD samples were markedly lower compared to those in normal colon tissues (Figure 4a). Importantly, we noted significant variations in NOL3 methylation levels across different genders. Additionally, we analyzed the methylation levels of NOL3 in normal tissues, adenocarcinoma, and mucinous adenocarcinoma, discovering significant differences among these categories (Figure 4b,c). To enhance our comprehension of the role of NOL3 methylation in COAD, we employed the SMART database. We initially presented the distribution of methylation probes associated with NOL3 across the chromosomes in COAD, followed by a comprehensive examination of the extensive genomic data linked to NOL3. Our investigation identified 16 methylation probes associated with NOL3 (Figure 4d). We then delved into the relationship between these methylation probes and the expression of NOL3 in COAD. Notably, we found a positive correlation with probe cg00332745, whereas other methylation probes displayed a negative correlation with NOL3 (Figure 4e). Lastly, we employed box plots to depict the expression differences of these methylation probes between COAD and normal samples, showing significant disparities for all probes except cg00332745 (Figure 4f). In conclusion, our analysis explored the relationship between NOL3 and various methylation probes in COAD, implying that NOL3 might play an oncogenic role in COAD through these methylation mechanisms.

### 3.5. Correlation Analysis of NOL3 and Immune Cell Infiltration Levels in COAD

**Figure 5:**
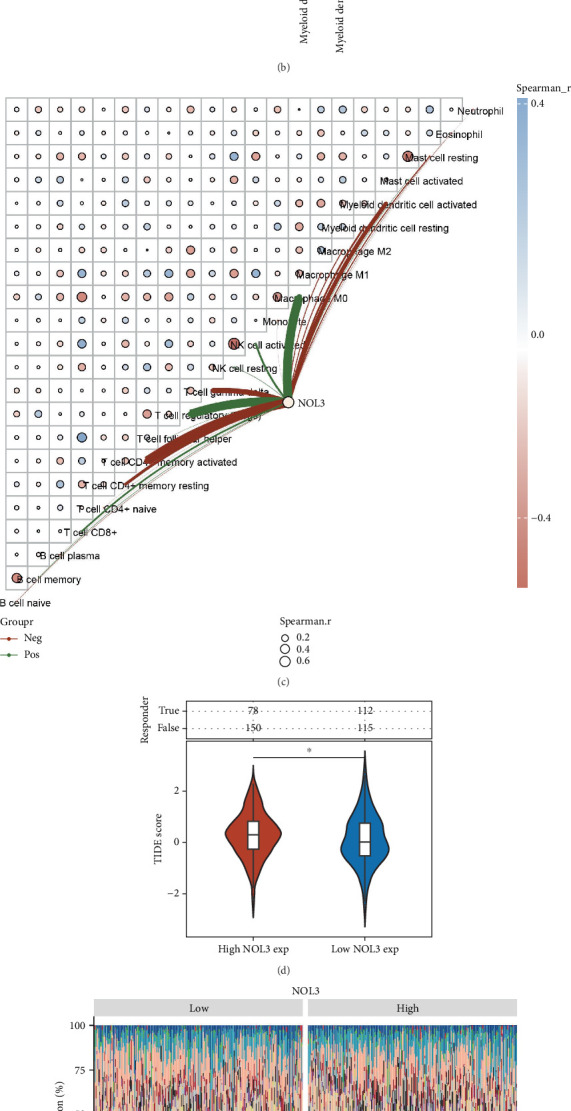
NOL3 is associated with immunotherapy for COAD. (a, b) Assessing the correlation between NOL3 and immune cell infiltration based on the CIBERSORT algorithm. (c) The correlation network diagram between NOL3 expression and immune score. (d) Analysis of NOL3 expression and response to immune checkpoint inhibitor therapy. (e) Immune cell scoring heatmap. (f) Analysis of the correlation between NOL3 and immune infiltration at the single-cell level. ⁣^∗^*p* < 0.05, ⁣^∗∗^*p* < 0.01.

Utilizing the CIBERSORT algorithm, we examined the variations in immune cell infiltration levels within COAD samples by dividing them into two groups based on NOL3 expression: high and low. Our findings revealed significant variations in the levels of several immune cell types, such as CD8+ T cells, memory resting CD4+ T cells, regulatory T cells (Tregs), gamma delta T cells, M0 macrophages, and activated myeloid dendritic cells, between the groups with high and low NOL3 expression (Figure 5a,b). In addition, we demonstrated the relationship between NOL3 expression and immune scores calculated from the CIBERSORT algorithm through corresponding network diagrams (Figure 5c). Following this, we explored how NOL3 expression impacted the effectiveness of immune checkpoint inhibitor therapy using the TIDE algorithm. The TIDE scores were significantly higher in the high NOL3 expression cohort when compared to the low NOL3 expression cohort, indicating that individuals with elevated NOL3 expression were likely to have a worse prognosis after receiving immune checkpoint inhibitor therapy (Figure 5d). Heatmaps illustrated the arrangement of various immune cells across the high and low NOL3 expression groups (Figure 5e). Lastly, we investigated the association between NOL3 expression and immune cell infiltration in COAD utilizing single-cell analysis (Figure 5f).

### 3.6. Analysis of the Correlation Between NOL3 and Chemotherapeutic Drugs in COAD

**Figure 6:**
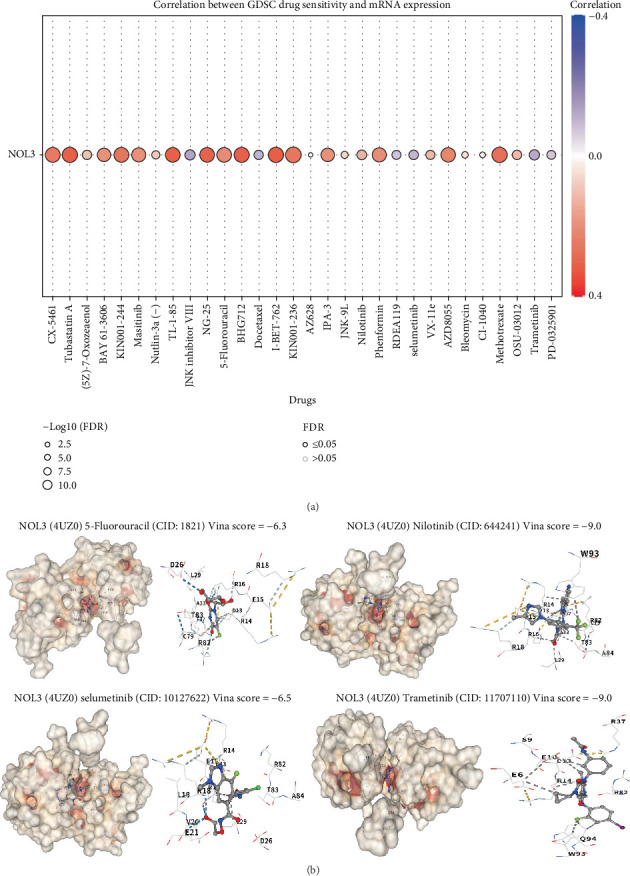
NOL3 is associated with chemotherapy drug sensitivity in COAD. (a) Correlation between GDSC drug sensitivity and NOL3 expression. (b) Molecular docking of NOL3 with chemotherapeutic agents.

To begin with, we investigated the relationship between NOL3 and a range of chemotherapeutic medications utilizing the GDSC database, illustrating the Top 30 drugs in a heatmap (Figure 6a). Our findings indicated that the majority of the chemotherapeutic agents displayed a positive correlation with NOL3, implying that elevated expression levels of NOL3 are associated with increased IC50 values for these agents. This observation implies that patients may show diminished sensitivity to these chemotherapeutic drugs. In addition, we evaluated the binding affinity of frequently used chemotherapeutics in COAD through molecular docking. Our findings revealed that NOL3 exhibited a significant binding affinity toward 5-fluorouracil, nilotinib, selumetinib, and trametinib (Figure 6b).

### 3.7. Knockdown of NOL3 Inhibits COAD Progression

**Figure 7:**
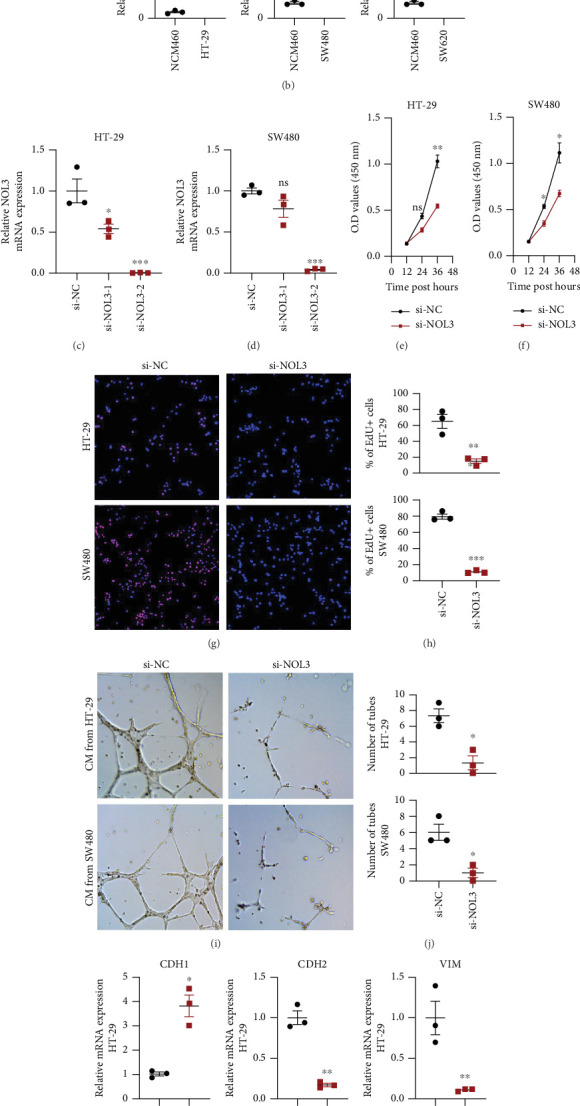
NOL3 enhances the progression of COAD. (a, b) The transcript levels of NOL3 were measured in CCD-841 CoN, NCM460, HT-29, SW480, and SW620. (c, d) Assessment of the efficacy of small interfering RNA in inhibiting NOL3. Following (e, f) the inhibition of NOL3, a reduction in cell viability was observed in COAD cell lines. (g, h) The proportion of EdU-positive COAD cells was decreased after NOL3 inhibition. (i, j) The angiogenic potential of COAD cell lines exhibited a decline subsequent to NOL3 inhibition. (k, l) The levels of EMT were found to be downregulated in COAD cell lines after NOL3 inhibition. ⁣^∗^*p* < 0.05, ⁣^∗∗^*p* < 0.01, ⁣^∗∗∗^*p* < 0.001.

To validate the function of NOL3 in COAD cells, we subsequently conducted in vitro functional experiments. Initially, we observed the presence of NOL3 mRNA in the colon cell lines CCD-841 CoN and NCM460, in addition to the COAD cell lines HT-29, SW480, and SW620 utilizing PCR methodologies. The levels of NOL3 mRNA were found to be markedly elevated in the COAD cell lines HT-29, SW480, and SW620 (Figure 7a,b). In light of this discovery, we developed two small interfering RNAs (si NOL3#1 and si NOL3#2) directed at NOL3 and assessed their effectiveness in reducing NOL3 expression in the HT-29 and SW480 cell lines. The si NOL3#2 demonstrated the most pronounced inhibitory effect on NOL3 and was employed in subsequent analyses (Figure 7c,d). Results from the CCK-8 assay indicated a significant decrease in cell proliferation following the application of si NOL3#2. Additionally, a notable reduction in the EdU-positive cell count was observed (Figures 7e, 7f, 7g, and 7h). We also explored the influence of NOL3 knockdown on the angiogenic potential of COAD cells. The data revealed that the capacity of the cells to form tubes was significantly impaired after introducing si NOL3#2 (Figure 7i,j). We examined the HT-29 cells through PCR following NOL3 suppression, as well as the expression levels of EMT-related markers in SW480 cells. Postinhibition of NOL3, there was a decrease in EMT markers (CDH1 was upregulated, while CDH2, VIM, and SNAIL were downregulated) (Figure 7k,l).

## 4. Discussion

COAD represents a highly aggressive form of cancer and has become one of the most common and lethal malignancies in the global digestive system [17]. The development of COAD is characterized by its complexity and arises from multiple factors, including genetic predispositions, environmental conditions, and lifestyle choices. A notable feature of COAD is its tendency toward invasiveness and a high potential for metastasis, irrespective of the varied underlying causes [18]. Often diagnosed at advanced stages, tumors typically invade adjacent tissues significantly or spread to other regions of the body, thereby complicating treatment options. While surgical interventions, chemotherapy, and radiation therapy can be effective during the initial stages, their impact tends to lessen as the cancer advances, with recurrence and metastasis emerging as major challenges in treatment. Furthermore, COAD cells demonstrate strong invasive and metastatic capabilities, largely driven by abnormal alterations in gene expression, signaling pathways, cellular migration, and their interactions with the surrounding microenvironment [19]. The complexity of these processes allows cancer cells to circumvent local barriers, gain access to the bloodstream or lymphatic systems, and disseminate extensively to distant sites such as the liver, lungs, and bones. These characteristics not only exacerbate the severity of the disease but also present significant challenges in achieving optimal outcomes with current treatment strategies. As a result, there is an urgent necessity for a more profound comprehension of the core mechanisms that govern COAD cell behavior, along with a thorough investigation into the biological foundations of their emergence, progression, invasion, and metastasis. By clarifying important signaling pathways, identifying gene mutations, recognizing epigenetic changes, and understanding the interactions within the tumor microenvironment, we can lay the groundwork for creating innovative, precisely targeted therapeutic strategies.

In recent times, advancements in technologies like genomics, proteomics, and single-cell sequencing have resulted in notable progress in comprehending the molecular pathological mechanisms associated with tumors. Nevertheless, numerous enigmas continue to be unsolved. Gaining a more profound insight into the cellular mechanisms that drive COAD is vital for creating more accurate diagnostic tools and tailored treatment strategies, thereby enhancing patient outcomes and lessening mortality rates. Our study highlights NOL3 as an essential regulator of COAD metastasis, as determined by WGCNA. NOL3 is essential in various tumor types. Research has demonstrated that the expression of NOL3 is significantly elevated in bladder cancer, where it promotes cell proliferation through the PI3K/Akt signaling pathway. Reducing NOL3 levels substantially inhibits the growth of bladder cancer cells, resulting in a stable cell cycle arrest in two distinct bladder cancer cell lines [20]. Furthermore, there is evidence to suggest that NOL3 exerts tumor-suppressive properties in myeloid tumors. The lack of NOL3 may trigger the development of myeloid proliferative tumors resembling primary myelofibrosis, with the underlying mechanism linked to the activation of the JAK-STAT pathway [21]. In the realm of the nervous system, NOL3 serves a protective function for neuronal cells against cell death induced by oxidative stress through the modulation of apoptosis pathways. Research indicates that the Tat-NOL3 protein can decrease the generation of reactive oxygen species (ROS), mitigate DNA fragmentation, and maintain mitochondrial membrane potential, thus safeguarding hippocampal neurons [22]. Moreover, NOL3 is also critical in the context of gastric cancer. The activation of the AKT protein during inflammation caused by *Helicobacter pylori* enhances telomerase activity, which is a key process in the development of gastric cancer [23]. NOL3 is thought to influence this mechanism by modulating the AKT signaling pathway. To summarize, NOL3 fulfills multiple roles across various tumor types, including enhancing cell proliferation, controlling apoptosis, and influencing signaling pathway regulation. These findings highlight the potential for NOL3 to emerge as a therapeutic target and offer new insights for future strategies in tumor treatment. However, the role of NOL3 in COAD has not yet been reported.

Our research identifies NOL3 as a pivotal gene in metastatic COAD, playing a significant regulatory role in patient prognosis, the immune microenvironment, and chemotherapy response among COAD patients. Gene enrichment analysis indicates that NOL3 is associated with DNA methylation in COAD. Aberrant DNA methylation is recognized as a promising biomarker with potential applications in the diagnosis, prognosis, and treatment of COAD [24–26]. DNA methylation represents one of the initially identified mechanisms for the epigenetic modification of genes, essential for sustaining normal cellular functions, maintaining chromosome integrity, facilitating X chromosome inactivation, regulating genomic imprinting, governing embryonic development, impacting aging processes, and contributing to cancer progression [27]. Tumors frequently show altered patterns of DNA methylation, typically marked by an overall decrease in methylation levels alongside localized increases in specific methylation areas. The state of hypomethylation can lead to the activation of oncogenes [28]. Our data suggest that the methylation level of NOL3 in COAD samples is significantly reduced compared to that in healthy colon samples. Consequently, we propose that the decline in NOL3 methylation might be associated with its role in advancing COAD metastasis. Subsequent in vitro experiments confirmed the impact of NOL3 on COAD metastasis.

This study has notable limitations. While it identifies key genes and related pathways derived from multiomics data and bioinformatics approaches, the predictive results necessitate comprehensive validation through additional in vivo and in vitro experiments to ensure the scientific accuracy of the research conclusions.

## 5. Conclusion

Our study identified NOL3 as a crucial gene in the metastasis of COAD through bioinformatics analyses and functional experiments. Notably, the knockdown of NOL3 significantly inhibited the progression of COAD.

## Data Availability

The data that support the findings of this study are available from the corresponding author upon reasonable request.
